# Analysis of immune-related key genes in Alzheimer’s disease

**DOI:** 10.1080/21655979.2021.1999553

**Published:** 2021-12-12

**Authors:** You Wu, Shunli Liang, Hong Zhu, Yaping Zhu

**Affiliations:** a The Second Clinical Medical College of Zhejiang Chinese Medical University, Zhejiang, P.R. China; b Department of Neurology, The Second Affiliated Hospital of Zhejiang Chinese Medical University, Zhejiang, P.R. China; c Department of Geriatric Psychiatry, Affiliated Mental Health Center & Hangzhou Seventh People’s Hospital,Zhejiang University School of Medicine, Zhejiang, P.R. China

**Keywords:** Alzheimer’s disease (AD), immune-related genes, weighted gene co-expression network analysis (WGCNA), immune-infiltration analysis, ROC curve analysis

## Abstract

This research revealed that 15 modules were obtained through weighted gene co-expression network analysis, among which the magenta and blue modules were significantly associated with Alzheimer’s Disease (AD). There were 121 genes in the magenta module and 1022 genes in the blue module. Through differently expressed genes analysis, significant differences were shown in 134 genes (88 were up-regulated and 46 were down-regulated). 34 immune-key genes were obtained after three types of genes were crossed. Functional enrichment analysis showed that genes were mainly enriched in cytokine receptor activity and immune receptor activity. Through protein–protein interaction (PPI) network analysis, 10 hub genes were obtained: *SERPINE1, ZBTB16, CD44, BCL6, HMOX1, SLC11A1, CEACAM8, ITGA5, SOCS3*, and *IL4R*. Through immune-infiltration analysis, significant differences were demonstrated in four immune cells: CD8 + T cells, resting NK cells, M2 macrophages, and activated dendritic cells, and a significant positive correlation was shown between CD8 + T cells and macrophages M2, or between the other two cells. CEACAM8 was positively correlated with CD8 + T cells and macrophages M2, and negatively correlated with the other two cells while the remaining nine genes showed the opposite. Receiver operating characteristic (ROC) curve analysis demonstrated that both the differential immune cells and 10 hub genes had good diagnostic values. In GSE122063, the hub genes were verified and *BCL6, CD44, HMOX1, IL4R, ITGA5,* and *SOCS3* were up-regulated. Meanwhile, hub genes was up-regulated in the brain tissues of AD rats. This study is of great significance for the diagnosis and therapy of AD.

## Introduction

Alzheimer’s disease (AD) is a neurodegenerative disease featuring memory loss, cognitive impairment, and personality alterations. It is the main cause of pre-senile and senile dementia. The disease was found by the German doctor Alois Alzheimer in 1906 [1]. The major pathological features of AD are senile plaque (SP) caused by the deposition of amyloid β (Aβ) in the brain, neurofibroustackles (NFT) resulted from intracellular over-phosphorylated τ (Tau) protein and anatrophic necrosis of neuronal cells [2–5]. The long course of AD imposes serious burdens to patients themselves, their families, and the society. At present, it is generally believed that AD is caused by genetic gene mutations and environmental predisposing factors that alter the normal metabolic process of amyloid precursor protein (APP), resulting in Aβ deposition to form SP. The abnormal phosphorylation of τ protein leads to NFT, resulting in neurodegenerative changes such as axonal transport, synaptic and mitochondrial dysfunction, and neuronal death, which in turn brings about cognitive impairment, mental and behavioral abnormalities, and other dementia symptoms [6,7]. Monsonego et al argued that Aβ contains both T lymphocytes and B lymphocyte locus, with the first 11 or 15 segments being B lymphocyte locus, and the 15–42 segments being T lymphocyte locus [8].

A study has found that innate immunity is necessary in AD pathogenesis and can become a driver for the disease progression [9]. During AD, neuroinflammation boosts the number of activated immune cells [10–12], and the T and B lymphocytes play major parts in the brain of AD patients [13]. Immune cells can also migrate from the blood and infiltrate into the brain in the inflammatory response [14]. Moreover, hyperpermeability of the blood–brain barrier enhances the infiltration of circulating immune cells [13,15]. It is known that during infiltration, T cells can generate interferon gamma (IFN-α), causing the accumulation of Aβ and consequently leading to cognitive dysfunction [16]. Currently, immunotherapy is crucial for the treatment of AD, but studies on immune-related genes are still very limited.

It is known that immune responses are important in AD pathogenesis, and we focus on identifying the immune key genes related to AD. In this study, the immune key genes related to AD were screened by bioinformatics analysis, and functional enrichment analysis was performed; meanwhile, the immune cells with significant differences were obtained through immune infiltration analysis; through receiver operating characteristic (ROC) curve analysis, the immune key genes, and differential immune cells screened in this study were found having good diagnostic values for AD. This study is of great significance for the diagnosis and therapy of AD.

## Materials and methods

### 1 Data download and preprocessing

With background correction and log2 normalization, GSE110226 was downloaded from the Gene Expression Omnibus (GEO) database based on the following criteria: (1) the brain tissue of AD patients, (2) the total number of samples >15 [17]. The gene expression profile was acquired from the tissues of the entire lateral ventricular choroid plexus after death. With six healthy samples set as controls, there were 4 with Frontotemporal dementia (FTD), seven with advanced AD and 3 with Huntington’s disease (HuD). GPL10379 platform [HuRSTA-2a520709] was used to conduct gene ID conversion. Another microarray data GSE122063 was also obtained from the GEO database [18]. Gene expression profiling was performed on the frontal and temporal cortex of 44 non-demented controls (Control) and 56 AD patients obtained from the University of Michigan Brain Bank. The ‘normalizeBetweenArrays’ of R package (R software version 3.6.3, https://www.r-project.org/) was used for the standardization of the expression data [19].

## 2 Weighted gene co-expression network analysis (WGCNA)

The variance of gene expression matrix was ranked from high to low, and the top 5000 genes were chosen for ‘WGCNA’ (R package) [20]. Modules and gene sets associated with clinical characteristics (age, gender, control, AD, FTD, and HuD) were found. In order to ensure the reliability of the co-expression network construction, abnormal samples were removed. A soft-threshold power, with scale-free *R*^2^ near 0.9 and slope near 1, was adopted to obtain a topological overlap matrix. The minimal module size of 30 was selected to build and detect the network. Modules with high correlation with AD were considered as key modules.

## 3 Identification of differential expression genes for AD

We analyzed the differently expressed genes (DEGs) between AD and the healthy samples using ‘limma’ in R package with adj.pvalue < 0.05 and | log2FC | ≥1 (FC, fold change) [19]. *P*-value was adjusted using Benjamini-Hochberg false discovery rate (BH-FDR). The volcanic map and heat-map images of DEGs were drawn.

## 4 Immune infiltration analysis

Immune-infiltration analysis was performed on both the AD and normal samples using ‘CIBERSORT’ in R package (*P* < 0.05) [21]. The immune cells that were not expressed in the samples were removed. Through the box plot, the differential immune cells between the AD and the controls were screened. The correlation analysis of 20 immune cells was carried out using R software.

## 5 Immune-related genes screening and functional enrichment analysis

4677 immune-related genes were obtained from the immPort immune database. The intersection genes of immune-related genes, key module genes obtained by WGCNA, and DEGs were collected. The immune-key genes were analyzed through Gene Ontology (GO) enrichment analysis (FDR<0.05) using ‘clusterProfiler’ in R package, and the functional roles of immune-key genes in the three aspects of biological processes (BP), cellular components (CC), and molecular functions (MF) were then predicted [22]. The biological pathway enrichment analysis was performed using FunRich (version = 3.1.3) to obtain the associated enrichment pathways [23].

## 6 The protein–protein interaction (PPI) network construction

PPI network was built based on the ‘Multiple Protein’ of the string database (http://string-db.org/, version 11.0), with a minimum required interaction score of 0.4 [24]. They were visualized by using the Cytoscape (version 3.8.2) software, and the hub genes were picked out through the use of cytoHubba.

## 7 Correlation analysis and ROC curve analysis of differential immune cells and hub genes

Correlation analysis was conducted to find out the relationship between hub genes and differential immune cells. The ROC curve analysis were performed using ‘pROC’ in R package to verify the diagnostic value of hub genes and differential immune cells [25].

## 8 Validation of hub genes

There were 44 controls and 56 AD patients in GSE122063, and the boxplot of hub genes expression was drawn using the ‘ggplot2’ in R package for validation.

## 9 Establishment of AD rat model

Healthy male Sprague-Dawley rats, weighing (250–280) g, were transported to the laboratory one week in advance for adaptive feeding. The rats with slow response or special sensitivity were eliminated, and the qualified rats were randomly divided into the Sham group and the AD group, with six rats in each group. After the rats were injected intraperitoneally with 10% chloral hydrate, aggregated Aβ_25-35_ was then injected into the bilateral hippocampus by a stereotaxic apparatus to construct the AD rat model. Morris water maze (MWM) was applied to evaluate the spatial learning and memory ability of rats [26]. MWM test is mainly divided into location and navigation test and space exploration test. The animal experiment protocol was approved by the Institutional Animal Care and Use Committee (IACUC), ZJCLA (ZJCLA-IACUC-20030017).

## 10 Quantitative real-time polymerase chain reaction (qRT-PCR)

The total RNA was isolated by using TRIzol reagent. The concentration was detected by NanoDrop Spectrophotometer (Thermos Fisher Scientific, USA), and mRNA underwent reverse transcription into cDNA with PrimeScriptTM RT reagent Kit (Takara, Japan). There was 20 μl qPCR reaction mixture: 10 μl SYBR® Premix Ex Taq II (Tli RNaseH Plus) (2×), 0.8 μl PCR Forward/Reverse Primer (10 μM), 0.4 μl ROX Reference Dye (50×), 80 ng DNA template and ddH2O. The qRT-PCR reactions were performed in the Applied Biosystems 7300 Real-Time PCR System (Bio-Rad, USA) as follows: pre-denaturation at 95°C for 30s; 40 cycles of denaturation at 95°C for 5s and annealing/extension at 60°C for 31s. β-actin acted as an internal reference gene. The relative expression levels of genes were calculated by 2^−ΔΔCT^ method. The primer sequences were showed in Table 1.

**Table 1.** The primer sequences used for qRT-PCR

Genes	GenBank	Forward primer	Reverse primer	Product sizes (bp)
BCL6	NC_051346.1	TAAGACTGGACCGAGGTTGT	CTCGCCTTCCCAGAAATGAA	98
CD44	NC_051338.1	CATGGAATTAAGGCCGAGCT	ATCTCAAACCACCACCCAAC	101
HMOX1	NC_051354.1	CATCCTCCAGCTCACATTCC	ACACCTGCCTAGACTGACTT	100
IL4R	NC_051336.1	AGGTGCTCAGAAAACTGTGG	GGTCAGAGGCCATGAAGAAG	99
ITGA5	NC_051342.1	GTGAAGCTGTTTTGGGAGGA	AAACGTCCACTTGAGCCATT	105
SOCS3	NC_051345.1	GCAGCTAATGAAACCTCCCA	CCATCTCCCCTTCTCAATGC	96

## 11 Western blot

The rats were decapitated and their brains were removed on the ice. The brain tissues of these rats were put into the glass homogenizer, and then lysate (Beyotime Biotechnology, China) was added, making it homogenized under the ice bath until it was fully cracked, and then the supernatant was obtained. BCA kit (Nanjing Jiancheng, China) was applied to detect the protein concentration. After that, the protein was separated by SDS-PAGE and transferred to the membrane. The PVDF membrane was placed in the blocking solution and incubated at room temperature for 1.5 h. The primary antibody (Invitrogen, USA) was added according to the instructions and incubated overnight at 4°C. The second antibody was added at the ratio of 1:5000 and incubated for 1.5 h. Finally, ECL was developed. The primary antibody: BCL6 (#PA5-27,390), CD44 (#PA5-21,419), HMOX1 (#MA1-112), IL4R (#PA5-36,394), ITGA5 (#711,210) and SOCS3 (#PA5-87,485).

## 12 Statistical Analysis

Results were displayed as the mean ± standard deviation ($\overset{ˉ}{x}$±*s*) and analyzed by using GraphPad Prism 8.0 software. Comparisons between the two groups were analyzed by Student’s *t*-test. For the MWM test, two-way repeated-measures ANOVA was used. *P* < 0.05 was considered to be statistically significant.

## Results

In this study, we aimed to identify the immune key genes related to AD. These genes were screened by WGCNA and DEGs analysis. Functional enrichment analysis was also performed and the immune cells with significant differences were obtained through immune infiltration analysis. Through ROC curve analysis, it was found that the immune key genes and differential immune cells screened in this study had good diagnostic values for AD. Finally, the expression of immune key genes was verified in another GEO database (GSE122063) and AD rat model.

## 1 WGCNA of GSE110226

**Figure 1.**
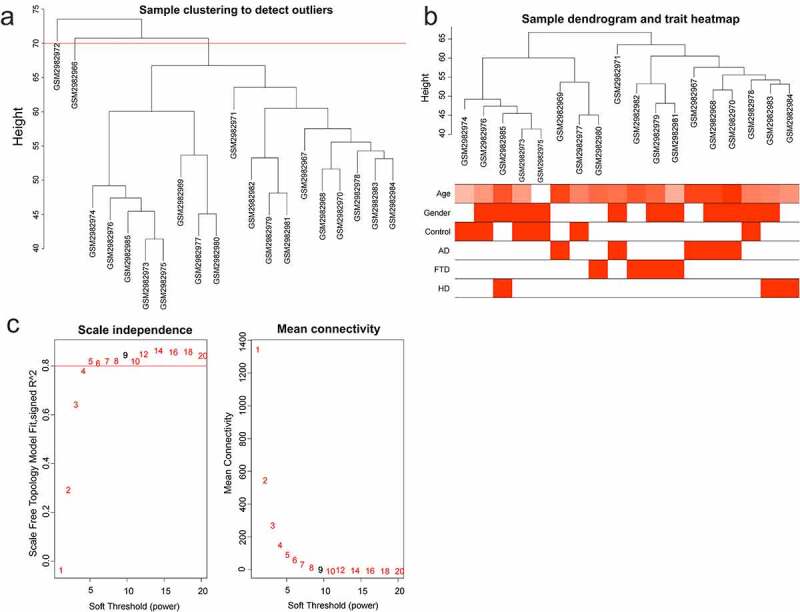
WGCNA of GSE110226. (a) Sample clustering tree. Two outlier samples were removed with a cut height of 70. (b) Sample dendrogram and trait heatmap. (c) The relationship between scale-free topology model fit or mean connectivity and soft-threshold power. The soft-threshold power was 9

**Figure 2.**
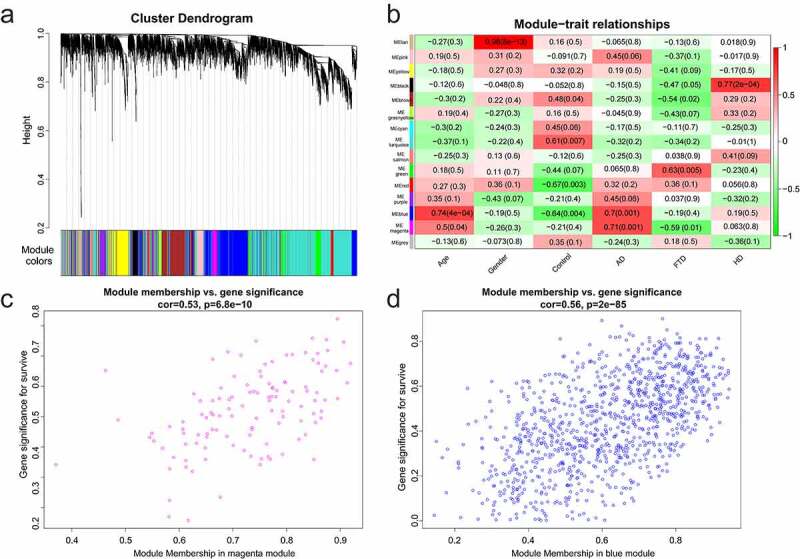
Module analysis. (a) Cluster dendrogram of gene. There were 15 modules, and different colors represented different modules. (b) Module–trait relationships. Red represented positive correlation and green represented negative correlation, and *P*-values were indicated in parentheses. (c) GS and MM in the magenta module. (d) GS and MM in the blue module

Data were normalized using ‘normalizeBetweenArrays’ to allow for more accurate subsequent analysis. Genes were sorted based on the variance of gene expression volume. The top 5000 genes were performed using WGCNA. Two outlier samples were removed with a cut height of 70 (Figure 1a). The samples were clustered, and the clinical characteristic heatmap was drawn (Figure 1b). To convert the adjacency matrix to a topological overlap matrix, the soft-threshold power was 9 (Figure 1c). There were 15 modules and their minimal module size was 30 (Figure 2a). A correlation analysis was conducted between the modules and the clinical features (age, gender, control, AD, FTD, and HuD) aiming to obtain modules significantly associated with AD: magenta and blue modules (Figure 2b). Furthermore, there was a positive correlation between AD and 2 modules. The relationship between gene significance (GS) and module membership (MM) was analyzed in two modules, with module correlation coefficient (cor) of 0.53 and 0.56 (*p* < 0.001) (Figure 2c-d). There are 121 and 1022 genes in the magenta and blue modules, respectively.

## 2 Differential gene analysis of GSE110226

**Figure 3.**
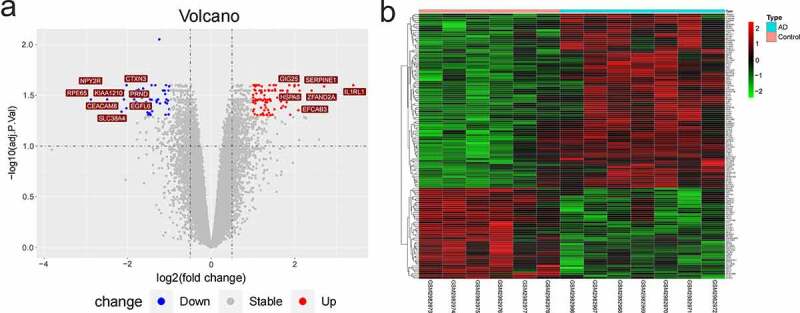
DEGs of GSE110226. (a) The volcanic map of DEGs. Red represented up-regulated genes and blue represented down-regulated genes. (b) The heat-map image of DEGs. Red represented up-regulation and green represented down-regulation

AD and normal samples were performed with differential gene analysis. Compared with the normal samples, there were 134 DEGs (88 up-regulated genes and 46 down-regulated genes). The volcanic map (Figure 3a) and heat-map image (Figure 3b) of DEGs were plotted. In the volcanic map, the following genes with | log2FC | >2: 6 up-regulated genes (*GIG25, SERPINE1, IL1RL1, HSPA6, ZFAND2A*, and *EFCAB3*) and eight down-regulated genes (*CTXN3, PRND, EGFL6, SLC38A4, CEACAM8, KIAA1210, NPY2R*, and *RPE65*).

## 3 The immune infiltration analysis in AD

**Figure 4.**
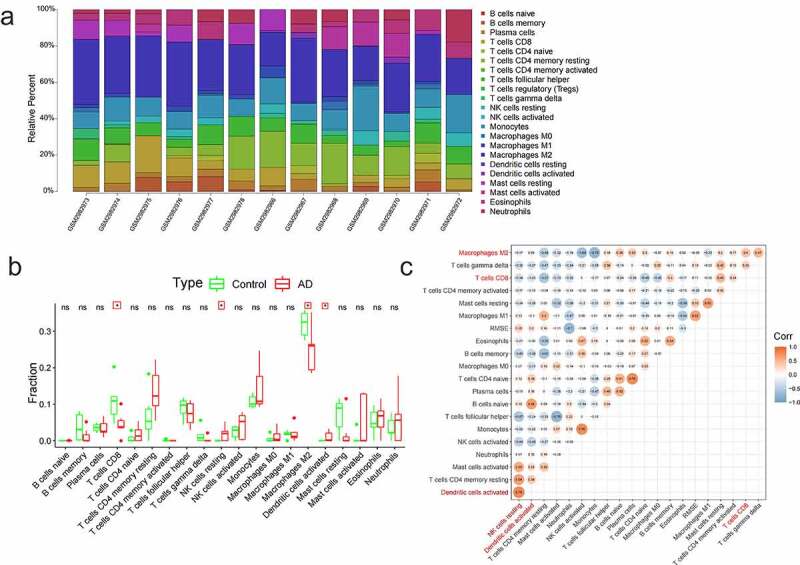
The immune infiltration analysis of AD. (a) The landscape of immune infiltration in AD and controls. Different colors represented different immune cells, and there were 22 immune cells. (b) The histogram of immune cells. There were four immune cells with significant difference (CD8 + T cells, M2 macrophages, activated dendritic cells, and resting NK cells). (c) The correlations analysis between immune cells. Orange represented positive correlation and blue represented negative correlation, and the digits represented Pearson correlation coefficient

Both the AD and normal samples were performed with the analysis of immune cells based on all gene expressions. Figure 4a showed the distribution of 22 immune cells (*p* < 0.05). Regulatory T cells and resting dendritic cells were not expressed in the samples and were therefore removed. Differential immune cells were displayed in the box plot diagram, and there were four immune cells with significant difference (CD8 + T cells, M2 macrophages, activated dendritic cells, and resting NK cells) (Figure 4b). The correlation analysis of 20 immune cells (Figure 4c) indicated that the strongest positive correlation was found between resting NK cells and activated dendritic cells (Pearson cor = 0.78), and the strongest negative correlation was found between mast cells activated and T cells follicular helper (Pearson cor = 0.75). It was also found out that there was a negative correlation between resting NK cells and CD8 + T cells or M2 macrophages (Pearson cor = 0.34, Pearson cor = 0.17); activated dendritic cells were negatively related to the CD8 + T cells (Pearson cor = 0.25), and weakly positively connected with the M2 macrophages (Pearson cor = 0.09); and CD8 + T cells were positively correlated with M2 macrophages (Pearson cor = 0.4).

## 4 Immune-related genes screening and functional enrichment analysis

**Figure 5.**
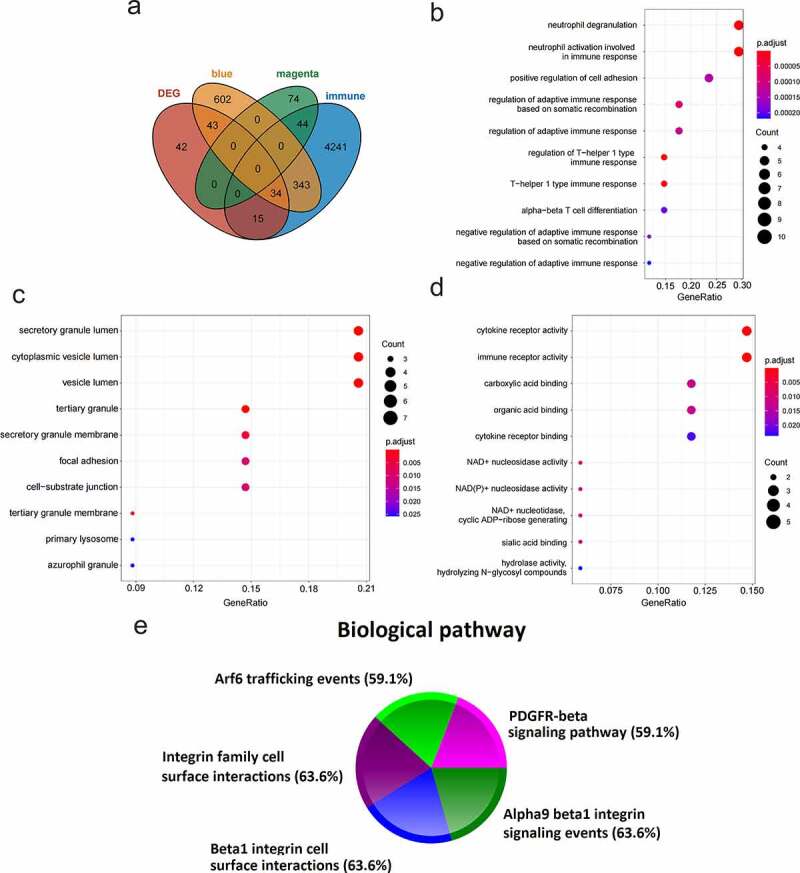
Functional enrichment analysis of immune-key genes. (a) The venn diagram of immune-related genes, key modules genes and DEGs. (b) GO-BP. (c) GO-CC. (d) GO-MF. (e) Biological pathway. Different colors represented different signaling pathways

4677 immune-related genes were obtained from the ImmPort immune database. 34 immune-key genes were obtained through the intersection of immune-related genes, two key module genes (magenta and blue modules), and DEGs (Figure 5a). GO enrichment analysis (GO-BP (Figure 5b), GO-CC (Figure 5c), GO-MF (Figure 5d)) and biological pathway analysis (Figure 5e) were performed on the immune-key genes. GO analysis manifested that these genes were mostly enriched in secretory granule lumen, and the neutrophil activation involved in immune response, cytokine receptor activity, immune receptor activity, etc. The analysis of biological pathway indicated that the genes were largely related to the following pathways: PDGFR-beta signaling pathway, integrin family cell surface interactions, beta1 integrin cell surface interactions, Arf6 trafficking events, and alpha9 beta1 integrin signaling events.

## 5 PPI network analysis

**Figure 6.**
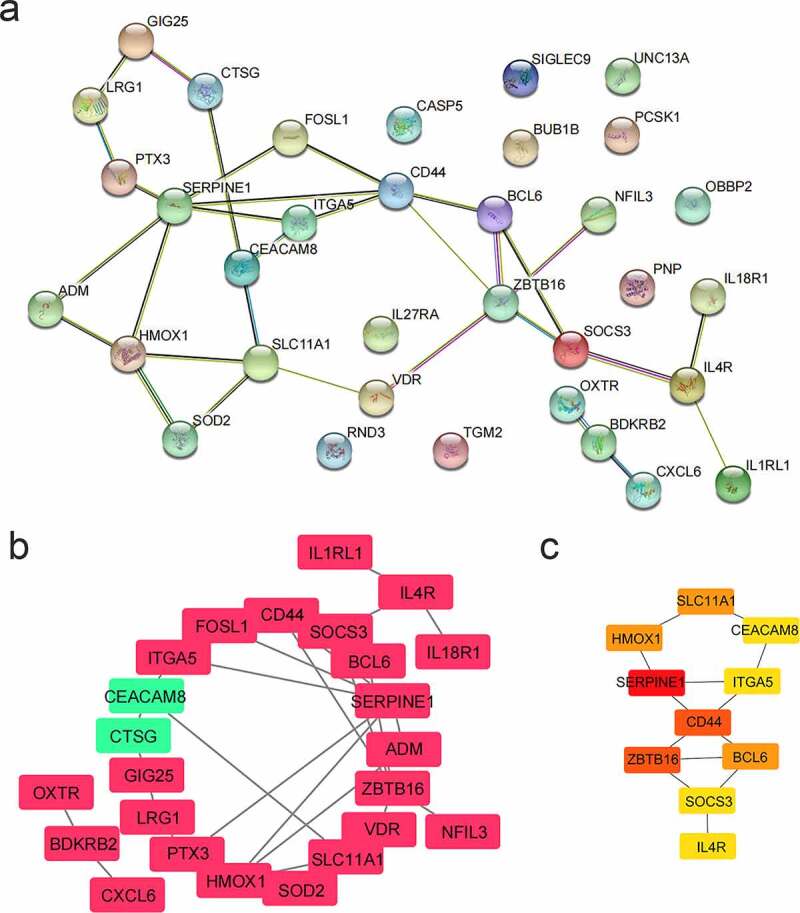
PPI network analysis. (a) PPI network. There were 34 genes. (b) PPI network visualization. Red represented up-regulated genes and green represented down-regulated genes. (c) Hub genes. According to the MCC algorithm, the top 10 genes were selected as hub genes. The redder the color, the higher the score of the hub genes

A PPI network for 34 genes was built (Figure 6a), and shown in Figure 6b (red represented up-regulated genes and green represented down-regulated genes). The top 10 genes were selected as hub genes based on the Maximal Clique Centrality (MCC) algorithm. The redder the color, the higher the score of the hub genes (Figure 6c). Hub genes were *SERPINE1, ZBTB16, CD44, BCL6, HMOX1, SLC11A1, CEACAM8, ITGA5, SOCS3*, and *IL4R*. Among them, the expression of *CEACAM* was down-regulated in AD patients, and the other nine genes were all up-regulated.

## 6 Correlation analysis of hub genes and immune cells

**Figure 7.**
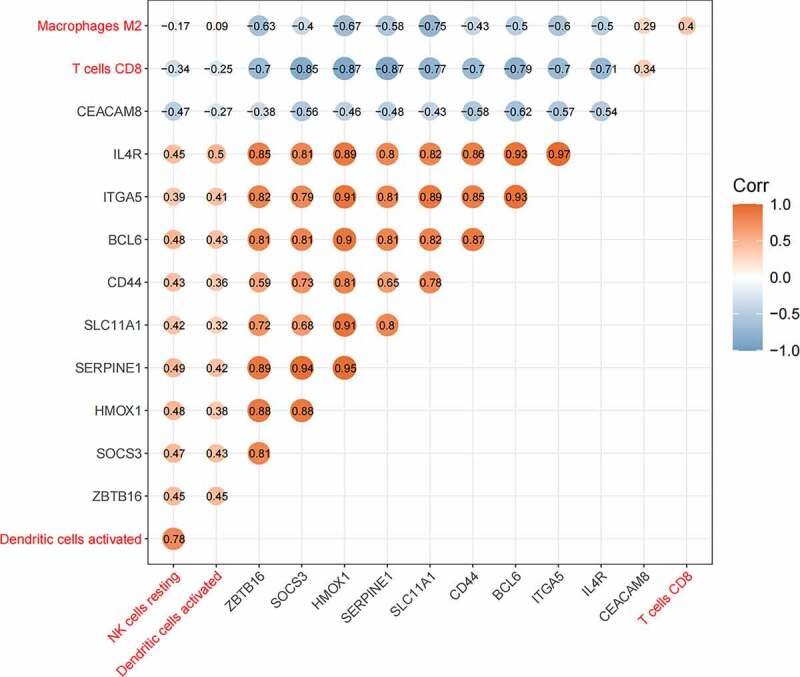
The relationship between hub genes and immune cells. Orange represented positive correlation and blue represented negative correlation, and the digits represented Pearson correlation coefficient

The relationship between hub genes and four kinds of immune cells (Figure 7) displayed that *CEACAM8* was positively correlated with macrophages M2 and CD8 + T cells, and negatively related to activated dendritic cells and resting NK cells. The other nine genes were positively related to activated dendritic cells and resting NK cells, and negatively correlated with CD8 + T cells and macrophages M2. *HMOX1* and *SERPINE1* had the strongest negative correlation with CD8 + T cells (Pearson cor = 0.87), and there was the strongest positive correlation between IL4R and activated dendritic cells (Pearson cor = 0.5).

## 7 The ROC curve analysis of hub genes and immune cells

**Figure 8.**
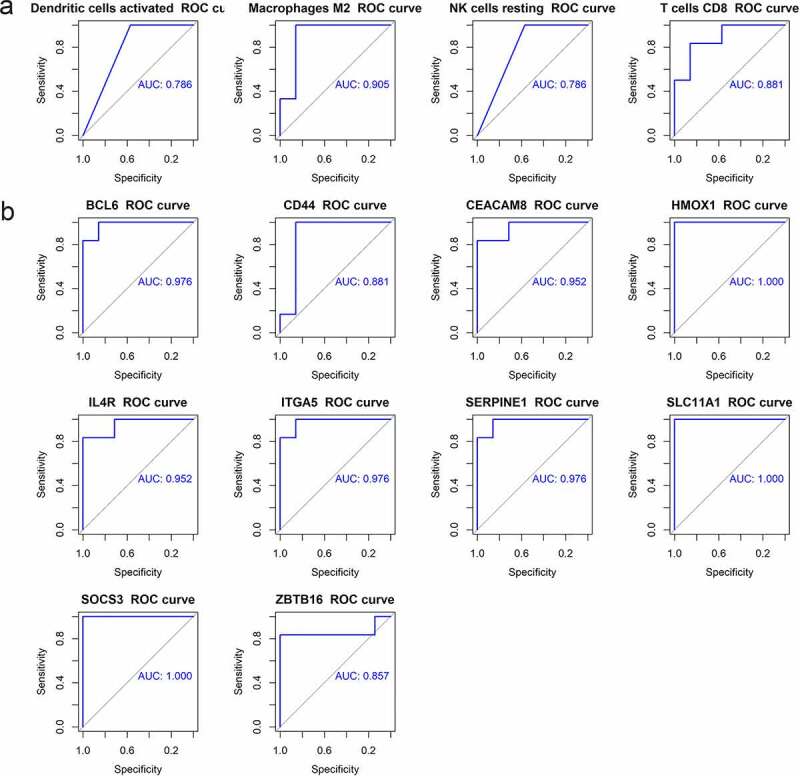
The ROC curve of hub genes and immune cells. (a) The ROC curve of four differential immune cells. (b) The ROC curve of 10 hub genes

ROC curves were analyzed to evaluate the diagnostic value of immune cells and hub genes for AD. ROC curve was drawn for four kinds of infiltrating immune cells, with the area under the curve (AUC) area greater than 0.75 (Figure 8a). ROC curves of 10 hub genes were shown in Figure 8b. Except for *CD44* and *ZBTB16*, whose AUC areas were 0.881 and 0.857, the AUC areas of all other genes were greater than 0.9. The above results showed that the four kinds of differential immune cells and 10 hub genes had good diagnostic values.

## 8 The validation of hub genes in GSE122063

**Figure 9.**
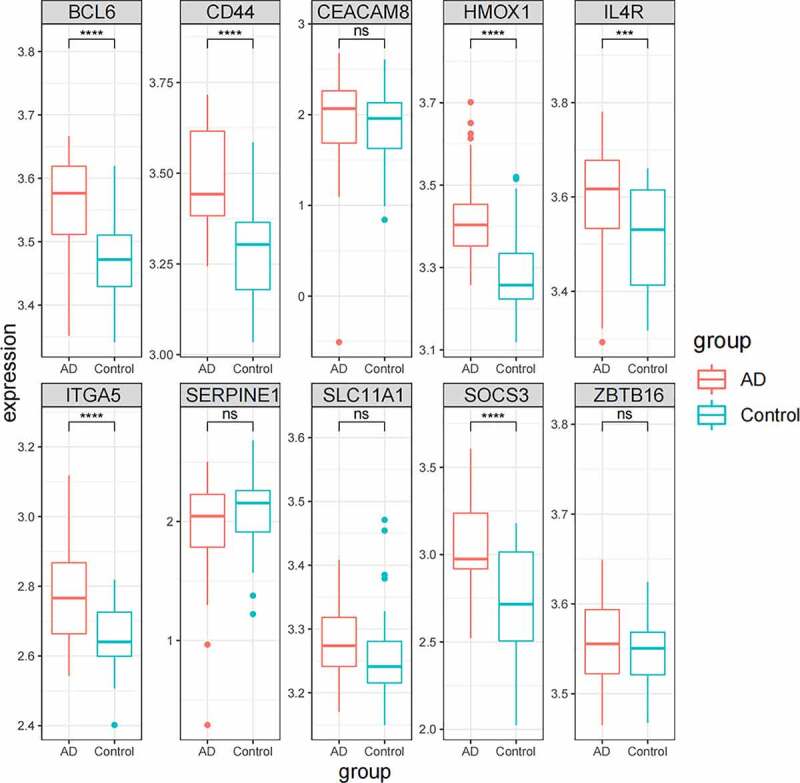
The expression of hub genes in GSE122063

In GSE122063, the expression levels of hub genes were verified. 44 normal controls and 56 AD patients were included in GSE122063. It could be seen in Figure 9 that the expressions of *BCL6, CD44, HMOX1 IL4R, ITGA5,* and *SOCS3* in AD patients were significantly up-regulated, which was consistent with GSE110226. There was no significant difference in *CEACAM8, SERPINE1, SLC11A1*, and *ZBTB16*.

## 9 Hub genes was up-regulated in AD rats

**Figure 10.**
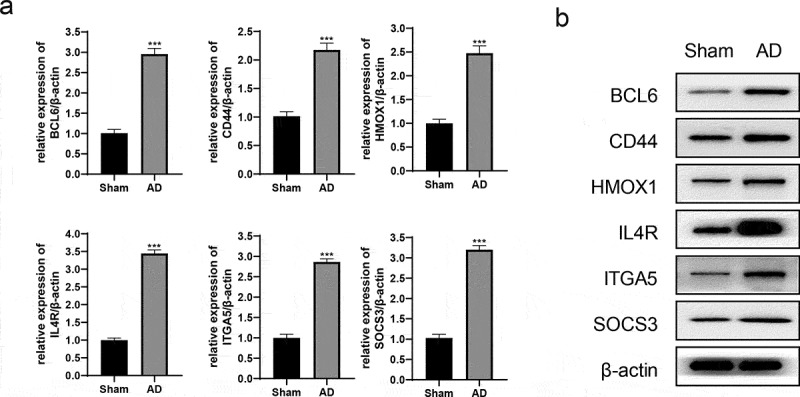
The expression of hub genes in AD rats. (a) The relative expression levels of hub genes/β-actin were detected by qRT-PCR. (b) The protein expression of hub genes was detected by western blot. ***P < 0.001

An AD rat model was established, and the MWM test showed that the model was successfully constructed. The results of location and navigation test (Table 2) indicated that the incubation period of AD rats was significantly longer than that of the Sham (68.37 ± 4.12 vs 28.96 ± 3.54, *P* < 0.05) and quadrant percentage was lower than that in the Sham (34.05 ± 3.19 vs 60.98 ± 2.56, *P* < 0.05). The results of space exploration test (Table 3) showed that the frequency of AD rats crossing the original platform within 2 min was significantly reduced (4.18 ± 0.45 vs 12.05 ± 0.33, *P* < 0.05) and the quadrant percentage decreased (29.64 ± 3.71 vs 59.61 ± 3.94, *P* < 0.05). After the model was successfully constructed, the rats were killed and their brain tissues (anterior temporal lobe and hippocampus) were removed. qRT-PCR results showed that the relative expression levels of hub genes mRNA were significantly up-regulated (Figure 10A). Meanwhile, the protein expression levels were also significantly up-regulated (Figure 10B). These results indicated that the expression of hub genes (*BCL6, CD44, HMOX1, IL4R, ITGA5,* and *SOCS3*) were up-regulated in AD rats, which were consistent with GSE110226 and GSE122063.

**Table 2.** Comparison of average incubation period and quadrant percentage of rats in location and navigation test ($\overset{ˉ}{x}$±*s*)

Group	n	Incubation period/s	Quadrant percentage (%)
Sham	8	28.96 ± 3.54	60.98 ± 2.56
AD	8	68.37 ± 4.12*	34.05 ± 3.19*

Note: The incubation period was the time when the rats found the platform within 2 minutes. Quadrant percentage is the percentage of swimming distance in the target quadrant of rats. Compared with the Sham, *P < 0.05

**Table 3.** Comparison of frequency of crossing the original platform and quadrant percentage of rats in space exploration test ($\overset{ˉ}{x}$±*s*)

Group	n	Frequency of crossing the original platform within 2 min	Quadrant percentage (%)
Sham	8	12.05 ± 0.33	59.61 ± 3.94
AD	8	4.18 ± 0.45*	29.64 ± 3.71*

Note: Quadrant percentage is the percentage of swimming distance in the target quadrant of rats. Compared with the Sham, *P < 0.05

## Discussion

Alzheimer’s disease, as a frequent cause of senile dementia, has resulted in serious medical, social, and economic problems worldwide. With the aging trend of population as a serious problem, the morbidity and mortality rate of AD is increasing, which poses harms and hinders on human health and social development [27]. AD is mainly caused by the abnormal metabolism of Aβ, which is produced by glycosylation APP cleaved by β-secretase and γ-secretase. In the physiological state, Aβ exists in the form of α-helix and has no neurotoxicity, but it can be transformed into toxic β-fold under the action of external factors, leading to fibrosis and amyloid deposition, and resulting in neurotoxicity [28,29]. In the process of AD, changes may occur in immune cells. It was reported that there were few T-cells in the brain of normal patients; nevertheless, this number was elevated in the brain of AD patients, especially in the hippocampus and temporal cortex, because of the disruption of the blood-brain barrier [30]. Besides, a study of AD reported that naïve T-cells and regulatory T-cells decreased, while memory T-cells and CD4 + T-cells increased compared with the control group [31,32].

In this study, we first conducted WGCNA on GSE110226 to obtain 15 modules (Figure 2a) and screened 2 key modules significantly related to AD (Figure 2b), namely the magenta module and the blue module. There were 121 and 1022 genes in the magenta and blue modules, respectively. Then, the differential expression gene analysis of the AD group and the control group in GSE110226 showed that there were 134 genes with significant difference, among which 88 were up-regulated and 46 were down-regulated. 4677 immune-related genes (from the ImmPort database), two key module genes (1143 genes, from WGCNA) and 134 DEGs were crossed to obtain 34 immune-key key genes (Figure 5a). GO analysis displayed that these genes were chiefly enriched in secretory granule lumen, neutrophil activation involved in the immune response, cytokine receptor activity, and immune receptor activity. Biological pathway analysis illustrated that genes were largely related to PDGFR-beta signaling pathway, integrin family cell surface interactions, beta1 integrin cell surface interactions, Arf6 trafficking events, and alpha9 beta1 integrin signaling events.

10 hub genes were screened through PPI network analysis and they were *SERPINE1, ZBTB16, CD44, BCL6, HMOX1, SLC11A1, CEACAM8, ITGA5, SOCS3*, and *IL4R*. It was reported that plasmin activation is negatively controlled by the fast-acting SERPINE1, leading to Aβ accumulation in the brain [33]. Karim et al found that *ZBTB16* may be related to neurodegenerative diseases [34]. Meanwhile, a study showed that *SOCS3* increased in areas with Aβ accumulation, demonstrating that *SOCS3* could adjust the central insulin signaling pathways in the AD [35].

The immune-infiltration analysis of GSE110226 illustrated significant differences between AD and healthy samples for four kinds of immune cells: CD8 + T cells, M2 macrophages, resting NK cells and activated dendritic cells (Figure 4b). The strongest positive correlation was found between resting NK cells and activated dendritic cells (Pearson cor = 0.78), and a negative connection was found between resting NK cells and CD8 + T cells or M2 macrophages (Pearson cor = 0.34, Pearson cor = 0.17); activated dendritic cells were negatively correlated with CD8 + T cells (Pearson cor = 0.25), and weakly positively related to M2 macrophages (Pearson cor = 0.09); CD8 + T cells were positively correlated to M2 macrophages (Pearson cor = 0.4). Adaptive immune cell populations have previously been reported to have a significant role in restraining AD pathology [36].

The correlations between hub genes and four kinds of immune cells (M2 macrophages, T cells CD8, activated dendritic cells, and resting NK cells) were analyzed (Figure 7). It was discovered that *CEACAM8* was positively correlated with CD8 + T cells and macrophages M2, and negatively related to resting NK cells and activated dendritic cells, while the other nine genes were inversely correlated. In this study, *CEACAM8* was found to be down-regulated in advanced AD, and the other nine genes were up-regulated; the number of CD8 + T cells and M2 macrophages was reduced, while the number of resting NK cells and activated dendritic cells increased. ROC curve analysis was used to assess the diagnostic value of hub genes and four immune cells, and it turned out that the AUC areas were all above 0.75, all of which had good diagnostic values.

The gene expression was verified in another GEO database (GSE122063). *BCL6, CD44, HMOX1, IL4R, ITGA5,* and *SOCS3* were significantly up-regulated in AD patients (Figure 9), which was consistent with GSE110226. Simultaneously, the AD rat model was constructed, and it was found that the expression of hub genes was up-regulated in the brain tissue of the AD rats.

## Conclusions

In this study, AD-related immune key genes with good diagnostic values were screened through bioinformatics analysis, which provided some clues for better understanding the potential molecular mechanisms of AD. However, these findings also need to be further verified by future experiments.

## Highlights

- There were two key modules significantly associated with AD through WGCNA in GSE110226.
- Immune infiltration analysis showed that there were four immune cells with significant difference.
- 34 immune-key genes were obtained through the intersection of immune-related genes, two key module genes, and DEGs. 10 hub genes were screened out through a PPI network.
- The correlation analysis and ROC curve analysis were carried out.
- AD rat model was constructed and the expression of hub genes were verified by qRT-PCR and western blot.
